# Comparison of the Reliability of Anatomic Landmarks based on PA Cephalometric Radiographs and 3D CT Scans in Patients with Facial Asymmetry

**DOI:** 10.5005/jp-journals-10005-1112

**Published:** 2011-04-15

**Authors:** Kamal Bajaj, Pooja Rathee, Pradeep Jain, Vasim Raja Panwar

**Affiliations:** 1 Reader, Department of Orthodontics, Rajasthan Dental College, Jaipur, Rajasthan, India; 2 Assistant Professor, Department of Orthodontics, Government Dental College, Jaipur, Rajasthan, India; 3 Professor and Head, Department of Orthodontics, Government Dental College, Jaipur, Rajasthan, India; 4 Clinical Assistant, Department of Orthodontics, Mahatma Gandhi Dental College, Jaipur, Rajasthan, India

**Keywords:** Frontal cephalometry, Lateral cephalometry, 3D CT scan.

## Abstract

**Introduction:**

Conventional cephalometry is an inexpensive and well-established method for evaluating patients with dentofacial deformities. However, patients with major deformities and in particular asymmetric cases are difficult to evaluate by conventional cephalometry. Reliable and accurate evaluation in the orbital and midfacial region in craniofacial syndrome patients is difficult due to inherent geometric magnification, distortion and the superpositioning of the craniofacial structures on cephalograms. Both two- and three-dimensional computed tomography (CT) have been proposed to alleviate some of these difficulties.

**Aims and objectives:**

The aim of our study is to compare the reliability of anatomic cephalometric points obtained from the two modalities: Conventional posteroanterior cephalograms and 3D CT of patients with facial asymmetry, by comparison of intra- and interobserver variation of points recorded from frontal X-ray to those recorded from 3D CT.

**Materials and methods:**

The sample included nine patients (5 males and 4 females) with an age range of 14 to 21 years and a mean age of 17.11 years, whose treatment plan called for correction of facial asymmetry.

All CT scans were measured twice by two investigators with 2 weeks separation for determination of intraobserver and interobserver variability. Similarly, all measurement points on the frontal cephalograms were traced twice with 2 weeks separation. The tracings were superimposed and the average distance between replicate points readings were used as a measure of intra- and interobserver reliability. Intra-and interobserver variations are calculated for each method and the data were imported directly into the statistical program, SPSS 10.0.1 for windows.

**Results:**

Intraobserver variations of points defined on 3D CT were small compared with frontal cephalograms. The intraobserver variations ranged from 0 (A1, B1) to 0.6 mm with the variations less than 0.5 mm for most of the points.

Interobserver variations of points between first and second tracings defined on PA Ceph and 3D CT were less than 1.5 mm for all the points.

A direct comparison of frontal cephalometry and 3D CT using intraobserver variation for the first observer showed that 3D CT was more reliable than frontal cephalometry for 11 points.

Direct comparison of frontal cephalometry and 3D CT for the second observer showed a similar tendency with 14 points significantly more reliable for 3D CT.

Comparison of frontal cephalometry and 3D CT using interobserver variation for the tracings showed that 3D CT was more reliable than frontal cephalometry.

**Summary and conclusion:**

The study has shown that 3D CT is consistently more accurate and reliable than conventional frontal cephalometry. Unfortunately, the cost of a computerized tomography examination is considerable; this means that, at this stage, its use by surgeons and orthodontists is a last resort.

## INTRODUCTION

Conventional cephalometry is an inexpensive and well-established method for evaluating patients with dentofacial deformities, and cephalometric evaluation in these patients has traditionally been performed by lateral and frontal cephalometry. These methods are well established due to the existence of a large normal-population databases. The cephalometric analysis is done by measuring lengths and angles defined by craniofacial reference points. Lateral cephalometry has proven valuable for the evaluation of patients with dentofacial deformities. In combination with frontal cephalometry, these methods are also applicable in patients with mild craniofacial asymmetries.

However, patients with major deformities and in particular asymmetric cases are difficult to evaluate by conventional cephalometry. Reliable and accurate evaluation in the orbital and midfacial region in craniofacial syndrome patients is difficult due to inherent geometric magnification, distortion and the superpositioning of the craniofacial structures on cephalograms. Both two- and three-dimensional computed tomography (CT) have been proposed to alleviate some of these difficulties.

Unlike conventional cephalograms, computerized tomography does not contain errors due to superposition of anatomic structures and differentiated enlargement in different areas. As a result, more accurate measurements have been reported on planar two-dimensional (2D) CT images. However, conventional CT depicts the head as a number of image slices instead of one image, and points measured on different CT image slices are difficult to evaluate. Both two- and three-dimensional CT have been reported to be accurate and reliable measuring methods. Studies with and without metallic markers showed that 3D CT to be better than 2D CT.

## AIMS AND OBJECTIVES

The aim of our study is to compare the reliability of anatomic cephalometric points obtained from the two modalities-conventional posteroanterior cephalograms and 3D CT of patients with facial asymmetry, by comparison of intra- and interobserver variation of points recorded from frontal X-ray to those recorded from 3D CT.

## MATERIALS AND METHODS

The subjects who participated in this study were drawn from the Department of Orthodontics, Government Dental College, Thiruvananthapuram, whose treatment plan called for correction of facial asymmetry. The sample included nine patients (5 males and 4 females) with an age range of 14 to 21 years and a mean age of 17.11 years. None had received previous orthodontic treatment. All the patients had full complement of teeth, including permanent second molars. Patients with known history of trauma resulting in facial asymmetry were excluded from the sample.

Computerized tomographic scans of head (without contrast) of the all the subjects were taken at the department of radio diagnosis, sree chitra tirunal institute for medical sciences and technology, Thiruvananthapuram. Scanning was done in a high speed CT/i GE medical systems spiral CT scanner (mAs 280, 3 mm slice thickness with 50% overlapping resulting in 1.5 mm effective slice thickness, 512 × 512 matrix, no tilt, pixel size 0.4 mm, standard head FOV of 25 cm). Axial scans parallel to the Frankfort horizontal (FH) plane were obtained, and CT data were transferred to a Silicon Graphics Workstation. CT image data were processed with a 3D program using a high-resolution bony reconstruction algorithm and displayed in SSD (shaded surface display). Window level/width was 263/1190 and the threshold values were defined as 200/3071 Hounsfield units (Hu) similar to standard values used for examination of bone in patients.

Frontal cephalograms (posteroanterior) were taken of the subjects at the Department of Oral Medicine and Radiology, Govt Dental College, Thiruvananthapuram, using a Planmeca Proline 2002 CC X-ray unit. Cephalograms of the subjects with teeth in centric occlusion were taken using a standardized cephalometric technique (78 kV, 12 mA, 1.2 second).

The head was fixed with ear posts with the head oriented in the Frankfort horizontal plane. The distance between transporionic axis and film was kept constant for each subject to minimize the magnification error. The central rays of X-rays passed through the center of the midsagittal plane so that the magnification of the right and left sides of the face was the same.

CT scan and frontal cephalogram of a skull were taken separately to find out the magnification factor. The magnification in CT (1 mm = 1.17 mm, 117%) and in frontal cephalogram (1 mm = 1.06 mm, 106%) was calculated and this magnification factor was employed to all CT scans and frontal cephalograms.

**Fig. 1:**
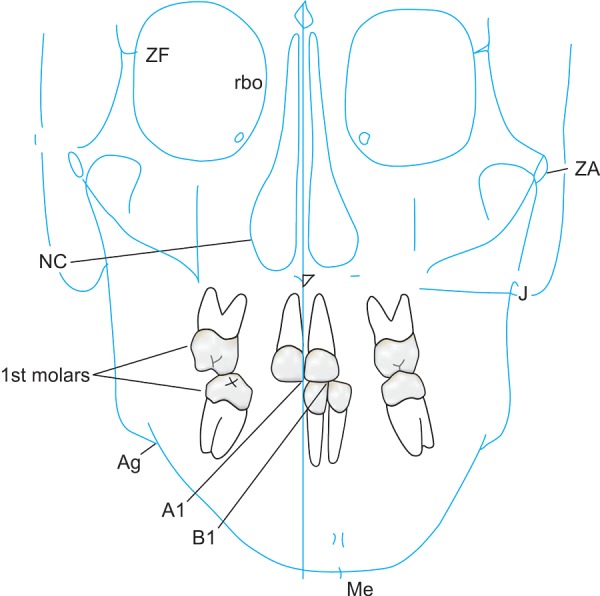
Cephalometric points considered

PA view cephalometrics was performed on both the CT scans and PA cephalograms. The cephalometric points considered were (Fig. 1) as follows:

1. ANS―anterior nasal spine
2. Me―menton (point of the inferior border of the sym-physis directly inferior to mental protuberance and inferior to the center of trigonium mentali)
3. NC (R)―nasal cavity at widest point (right)
4. NC (L)―nasal cavity at widest point (left)
5. Z (R)―zygomaticofrontal suture medial aspect (right)
6. Z (L)―zygomaticofrontal suture medial aspect (left)
7. MO (R)―medial orbit (right)
8. MO (L)―medial orbit (left)
9. Or (R)―orbit lower border (right)
10. Or (L)―orbit lower border (left)
11. UM (R)―upper molar (right)―the most prominent lateral point on the buccal surface of maxillary right first molar
12. UM (L)―upper molar (left)―the most prominent lateral point on the buccal surface of maxillary left first molar
13. LM (R)―lower molar (right)―the most prominent lateral point on the buccal surface of mandibular right first molar
14. LM (L)―lower molar (left)―the most prominent lateral point on the buccal surface of mandibular left first molar
15. A1 ―upper central incisor edge
16. B1 ―lower central incisor edge
17. Ag (R)―right antegonial notch
18. Ag (L)―left antegonial notch
19. J (R)―right jugal process
20. J (L)―left jugal process.

**Fig. 2:**
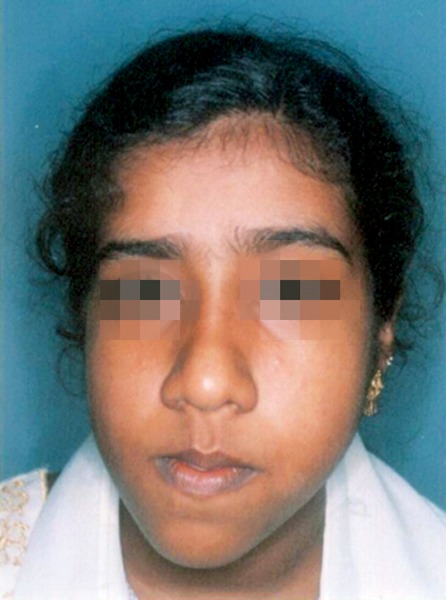
Typical patient

**Fig. 3:**
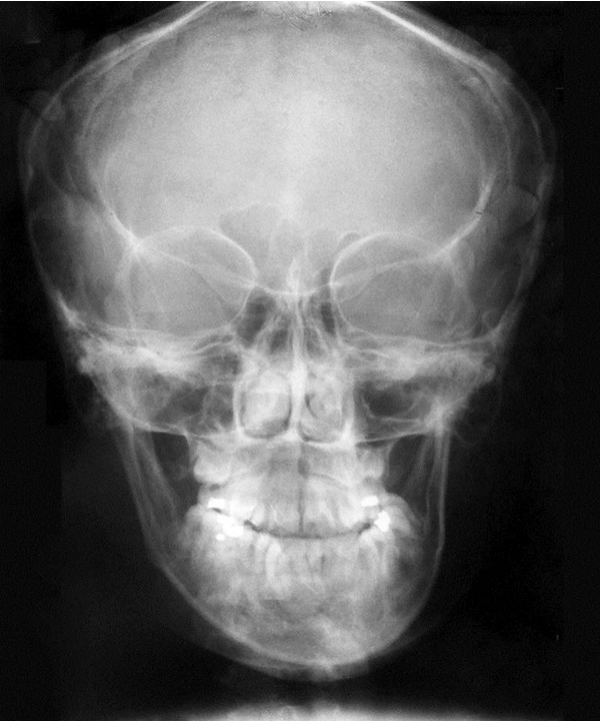
PA ceph of typical patient

**Fig. 4:**
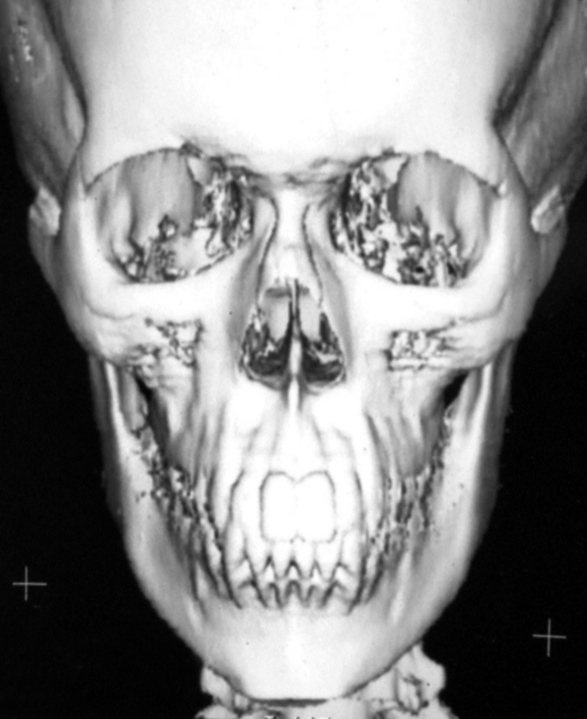
3D CT of typical patient

**Fig. 5:**
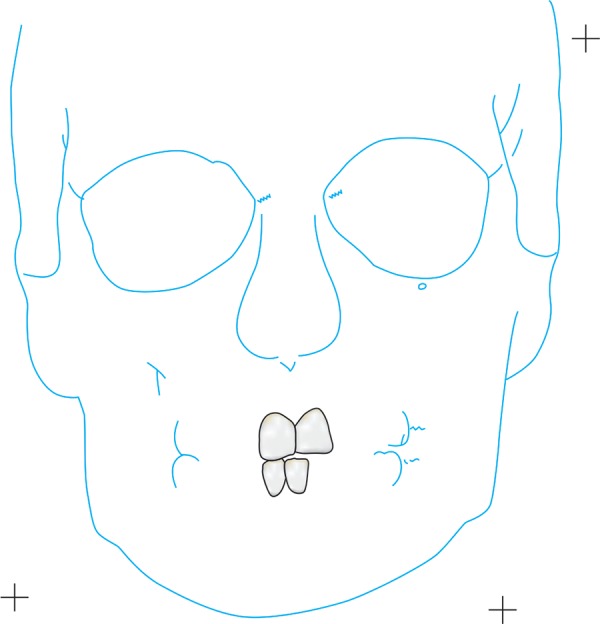
First tracing of 3D CT

**Fig. 6:**
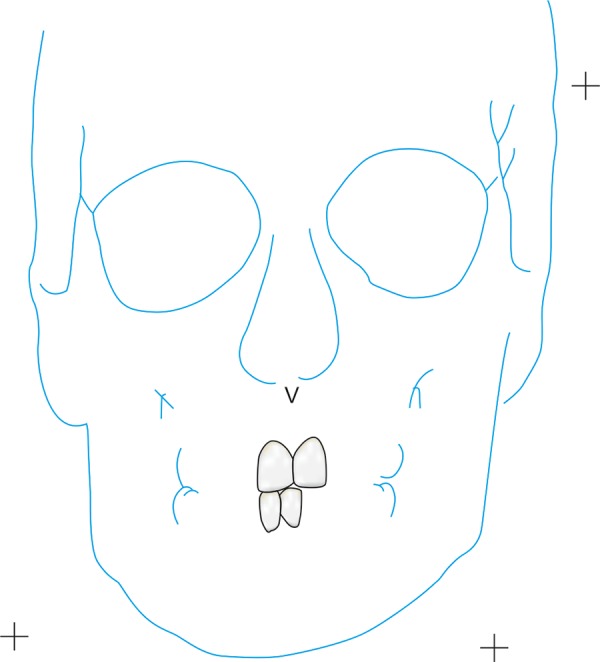
Second tracing of 3D CT

All nine CT scans were measured twice by two investigators with 2 weeks separation for determination of intraobserver and interobserver variability. Similarly, all measurement points on the frontal cephalograms were traced twice with 2 weeks separation (Figs 2 to 6). The tracings were superimposed and the average distance between replicate point readings were used as a measure of intra- and interobserver reliability. Intra- and interobserver variations are calculated for each method and the data were imported directly into the statistical program, SPSS 10.0.1 for windows.

For each variable, mean, standard deviation and standard error of the mean were calculated. The values are tested for statistical significance using the independent-samples t-test procedure. The level of significance was set at 0.05.

## RESULTS

**Fig. 7:**
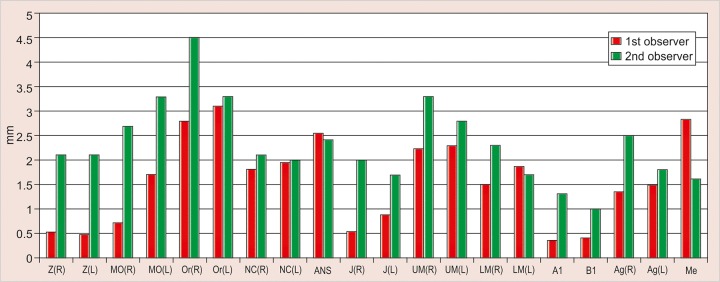
Intrainvestigator point variation on PA ceph

**Fig. 8:**
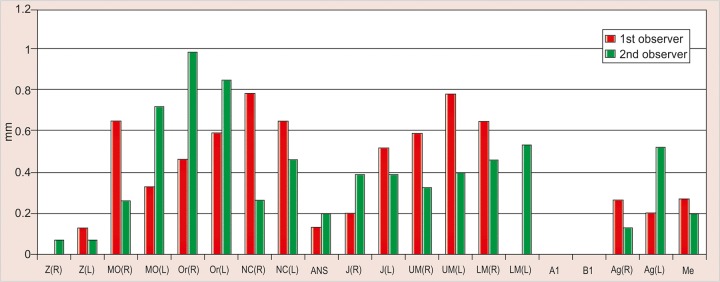
Intrainvestigator point variation on 3D CT

**Fig. 9:**
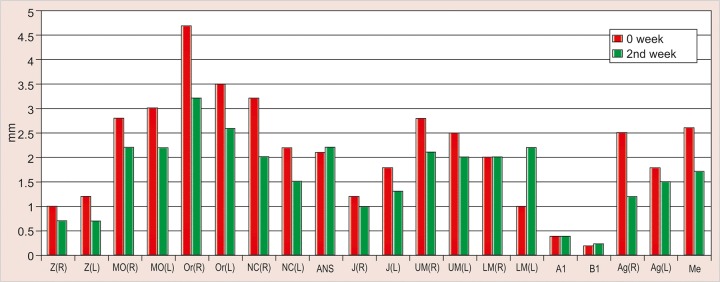
Interinvestigator point variation on PA ceph

**Fig. 10:**
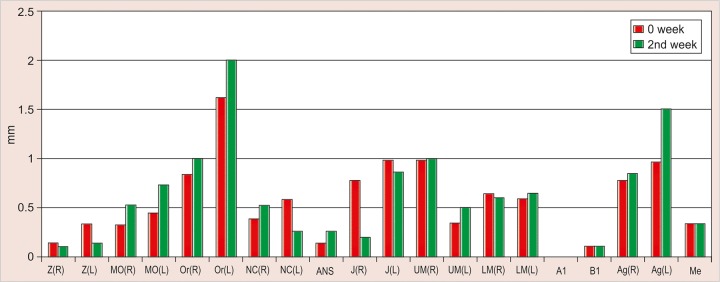
Interinvestigator point variation on 3D CT

The values obtained on superimposing the tracings were recorded in a suitable proforma. The tracings were superimposed and the average distance between replicate point readings were used as a measure of intra- and inter-observer reliability. Intra- and interobserver variations are calculated for each method (3D CT and frontal cephalograms) and the observations are shown in Figures 7 to 10.

**Fig. 11:**
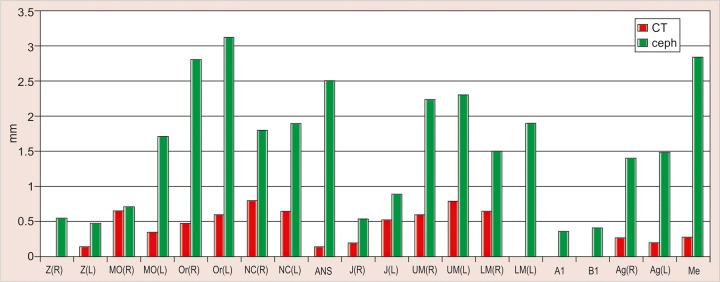
Intrainvestigator variation between PA ceph and 3D CT (1st investigator)

**Fig. 12:**
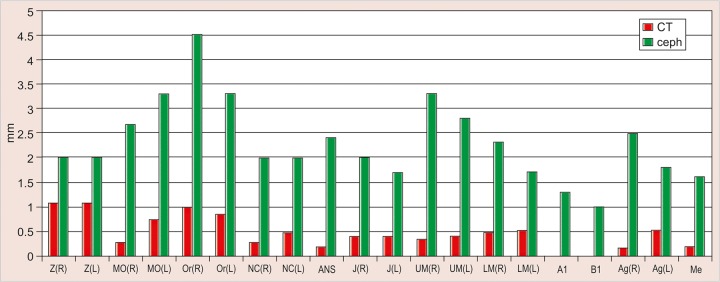
Intrainvestigator variation between PA ceph and 3D CT (2nd investigator)

**Fig. 13:**
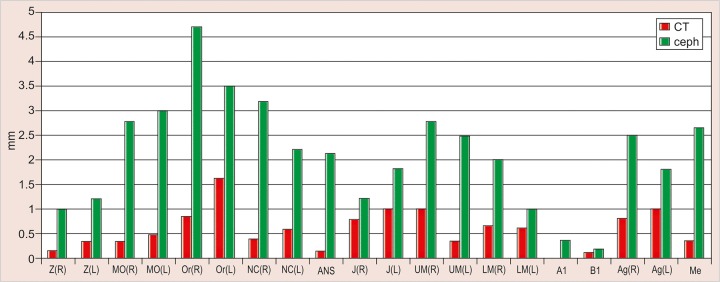
Interinvestigator variation between PA ceph and 3D CT (Ist tracing)

**Fig. 14:**
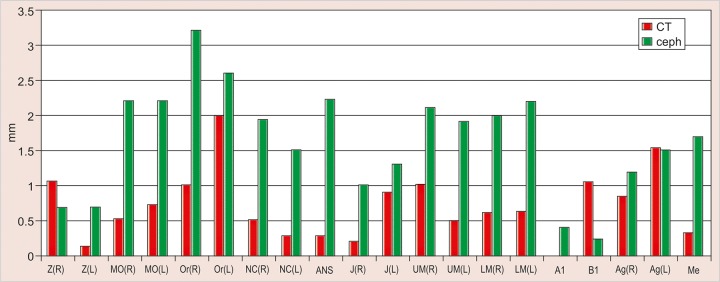
Interinvestigator variation between PA ceph and 3D CT (2nd tracing)

Intraobserver point variations between PA Ceph and 3D CT for the first observer and the second observer were calculated. Similarly, interobserver point variations between PA Ceph and 3D CT for the first tracing and the second tracing were also calculated. Figures 11 to 14 show the comparison of the intra- and interobserver point variation between PA Ceph and 3D CT.

Statistical analysis was performed using a statistical package SPSS version 10.0 for Microsoft Windows in a Pentium III computer. Routine statistical functions, like mean, maximum and minimum, range, standard deviation and standard error, were calculated for each group. The values are tested for statistical significance using the independent-samples t-test procedure. The level of significance was set at 0.05.

Intraobserver variations of points defined on 3D CT are shown in Figure 8. The variations were small compared with frontal cephalograms. The intraobserver variations ranged from 0 (A1, B1) to 0.6 mm, with the variations less than 0.5 mm for most of the points. The only statistically significant difference between the two investigators was observed for measuring of the left lower molar (LML) ( p < 0.05).

Interobserver variations of points between first and second tracings defined on PA Ceph are shown in Figure 9. The interobserver variations were less than 1.5 mm for all the points. No statistically significant differences were observed with any of the points. Interobserver variations of points between first and second tracings defined on 3D CT are shown in Figure 10. The variations were small compared with frontal cephalograms and no statistically significant differences were observed with any of the points.

A direct comparison of frontal cephalometry and 3D CT using intraobserver variation for the first observer (see Fig. 11) showed that 3D CT was more reliable than frontal cephalometry for 11 points, Or (R), Or (L), ANS, UM (L), LM (L), Me (p < 0.001, very highly significant), MO (L), NC (L), Ag (L) (p < 0.01, highly significant), UM (R) and Ag (R) (p < 0.05, significant). Direct comparison of frontal cephalometry and 3D CT for the second observer (see Fig. 12) showed a similar tendency with 14 points significantly more reliable for 3D CT Z (R), Or (R), ANS, UM (R), UM (L), A1 (p < 0.001), Z(L), MO (R), MO (L), Or (L), B1, Ag (R) (p < 0.01), J(R) and LM (R) (p < 0.05).

Comparison of frontal cephalometry and 3D CT using interobserver variation for the first tracings (see Fig. 13) showed that 3D CT was more reliable than frontal cepha-lometry for 12 points, Or (R), NC (R) (p < 0.001), MO (R), MO(L), ANS, UM (L), LM (R), Me (p < 0.01), Or (L), NC (L), UM (R) and Ag (R) (p < 0.05). Comparison of frontal cephalometry and 3D CT using interobserver variation for the second tracings (see Fig. 14) showed a similar trend with 3D CT more reliable than frontal cephalometry for 10 points, ANS (p < 0.001), MO (R), Or (R), LM (R), Me (p < 0.01), MO(L), NC (R), NC (L), UM (L), and LM (L) (p < 0.05).

## DISCUSSION

Conventional cephalometry is an inexpensive and well-established method for evaluating patients with dentofacial deformities. Since the introduction of cephalometric radiography, orthodontists have focused on the lateral cephalo-grams as their primary source of skeletal and dentoalveolar data; however, posteroanterior (PA) cephalometric projections and relevant analyses constitute an important adjunct for qualitative and quantitative evaluation of the dentofacial region. Lateral cephalometry, in combination with frontal cephalometry, is applicable in patients with mild craniofacial asymmetries but provides little useful information on asymmetries in ramal height, mandibular length and gonial angle. It is limited by the fact that the right and left structures are superimposed on each other and are at different distances from the film and X-ray source resulting in significant differences in magnifications.^1^

The posteroanterior (PA) cephalogram contains diagnostic information not readily available from other sources. This information allows the practitioner to evaluate the width and angulation of the dental arches in relation to their osseous bases in the transverse plane; evaluate the width and transverse positions of the maxilla and mandible; evaluate the relative vertical dimensions of bilateral osseous and dental structures; assess nasal cavity width; analyze vertical and/ or transverse facial asymmetries. The frontal radiograph is a valuable tool for comparing right and left structures because they are located at relatively equal distances from the film and radiographic source.^2^ Gross inspection of PA cephalogram can provide useful information concerning overall morphology, shape and size of the skull, bone density, suture morphology and possible deviations from these. Further, it can contribute to the detection of pathology of the hard and soft tissues.

Studies have shown that the PA film to have some inherent limitations of methodology and reliability. Baumrind and Frantz^3^ pointed out that there are two general classes of errors associated with cephalometric measurements. The first class of errors is ‘projection’ errors, which result because the cephalograph is a two-dimensional image of a three-dimensional subject and arise from the geometry of the radiographic set-up. The second general class of landmark errors may be termed ‘errors of identification’ and arise due to uncertainty involved in locating specific anatomic landmarks on the radiograph.

Accurate landmark identification is the single most important prerequisite for recording valid measurements. The errors in the location of landmarks could be due to the inherent placement of the landmark identification by the investigator, i.e. systemic error. Other errors could be in the reproducibility of the same landmark by the same individual, i.e. intraobserver error. The errors in the measurement can also be due to variation in identification of the same landmark by different individuals and also in making measurements, called interobserver errors. The intrainvestigator errors can be minimized with experience and practice. The intra- and interinvestigator errors for scientific purposes need to be calculated using Dahlberg’s formula and Midtgard’s method. To be scientifically acceptable, the error of variance should be close to 3%.^4^

El-Mangoury et al^5^ determined the horizontal, vertical and radial variability of 13 landmarks in posterior anterior cephalometrics. They found that each landmark had its own characteristic noncircular envelope of error, and that the variability is different in the horizontal and vertical directions. Major et al^6^ conducted a study which was designed to quantify the intraexaminer and interexaminer reliability of 52 commonly used posterior anterior cephalometric landmarks. The horizontal and vertical identification errors were determined for a sample of 33 skulls and 25 patients. The results showed that there is a considerable range in the magnitude of error with different horizontal and vertical values. Interexaminer landmark identification error was significantly larger than intra-examiner error for many landmarks. The identification error was different for the skull sample compared with the patient sample for a number of landmarks. In general, landmarks are less reliable on patient radiographs where soft tissue reduces hard tissue image sharpness.

Reliable and accurate evaluation in the orbital and midfacial region in craniofacial syndrome patients is difficult due to inherent geometric magnification, distortion and the superpositioning of the craniofacial structures on cephalograms.^7-9^

Due to the inherent limitations of methodology and reliability, conventional cephalometric has produced some questions concerning the validity of this scientific method.^10-12^ Further, the inadequacy of some cephalometric methods has led some authors to reject entirely conventional cephalo-metric analysis and to suggest the adoption of mathematical and engineering techniques for description of change in form.^12-14^

The submental vertex (SMV) view has been suggested as a better alternative for the cephalometric assessment of asymmetry; however, the SMV view is capable of significant distortion, especially in the analysis of mandibular asymmetry, since the mandible is positioned farthest from the film plane. While still useful in comparative research studies of asymmetry, both PA and SMV roentgenograms may have limited value in orthodontic diagnosis and treatment planning for the individual.^15^

Three-dimensional radiography by either CT scans or the digitized integration of sagittal and posteroanterior cephalograms probably offers the most promise today in the analysis of multiplane skeletofacial deformity, including asymmetry. CT scans avoid the superimposition of structures and differentiated enlargement problems, and therefore, more desirable than conventional radiography as a morphometric tool. As a result, more accurate measurements have been reported on planar two-dimensional (2D) CT images.^9,16^ However, conventional CT depicts the head as a number of image slices instead of one image, and points measured on different CT image slices are difficult to evaluate.

There are several other problems associated with CT. Window setting, partial-volume effects, spatial uniformity and resolution, scan noise and artifacts can influence the quality of the CT images and subsequent 3D image reconstruction. ^16^ Partial-volume averaging phenomena is seen when a CT volume element (voxel, 0.5 × 0.5 × 2 mm) is partly filled with a dense material, like bone (typical 1000 to 2000 HU) and less dense material, like muscle (40 HU) or air (-1000 HU), giving rise to voxel values of, for instance, 800, 500, 100 or 0 HU. If the resulting averaged value is 500 to 800 HU, voxels appear completely filled with bone. If the value is zero (50% bone and 50% air), the voxels appear filled with water which has the X-ray attenuation value of 0 HU. The result will be a hole in the 3D skull filled with water (i.e. a pseudo foramen). By changing the 3D threshold, it is possible to include such an averaged voxel or to exclude it. Thin bony structures, like ethmoidal and nasal bones, account for only a small fraction of the total volume of a voxel, causing the averaged value to be between 0 and -1000 HU. Exclusion of such voxels by choosing a high threshold level gives rise to pseudoforamina in the 3D model, while inclusion of all averaged voxels causes the bone margins to be thicker than in reality.^9^

In the literature, the accuracy and reproducibility for linear measurements in 2D CT are reported to be excel-lent,^16^ but the use of metallic markers has significantly influenced the ease with which points could be defined. 2D CT measurements have been shown to be inferior to 3D CT measurements when points are taken from different CT slices,^17^ while measurements made on the same slice have been reported to be accurate and reliable.^16^ In addition, problems arise when points, such as A point, are defined on structures outside the scan plane on a normal transversal CT scan. In contrast, defining a point on a 3D CT image reconstruction is relatively easy. Again, however, it must be emphasized that it is only, at least in part, a mathematically interpolated model of the true anatomy.

The points recorded in this study are standard points for conventional cephalometry that are optimal for this imaging modality,^3,4,18-24^ and the reliability of these points has been well established. Studies have found that 3D CT to be better than 2D CT,^17,25^ but these studies were based on the use of metallic markers. The use of metallic markers will transform these skull studies to phantom studies. Phantom studies will only show how accurate the equipment is in obtaining the values and do not address the clinical problem of identifying anatomic landmarks.

The study by Hildebolt et al^17^ has dealt with validation of 3D CT without the use of metallic markers. This anthropometric study compared 3D CT length measurements with caliper measurements, measurements obtained from 3D digitizer and measurements made directly on the 2D slices. The coordinates of 22 landmarks used in the study were used only to calculate 26 length measurements and do not estimate the reliability and accuracy of the points.

Kragskov J et al^9^ compared the reliability of anatomic cephalometric points from conventional cephalograms and 3D CT, using nine human dry skulls, without metallic markers. Intra- and interindividual variations were calculated for each method and tested for statistical significance. They concluded that the benefit of 3D CT cephalometric is indicated to be in the severe asymmetric craniofacial syndrome patients, as conventional cephalometrics is known to be inferior in these cases.

The present study was also without the use of metallic markers and has shown that 3D CT is consistently more accurate than conventional frontal cephalometry. Intra-observer variations of points defined on 3D CT were small compared with frontal cephalograms. The intraobserver variations of points on frontal cephalograms varied between 0.1 to 1.75 mm. The variation was less than 1 mm for most of the points except Z (R) and Z (L) (right and left zygomatico frontal suture), Or (R) (right orbitale), J (R) (right J point) and A1 (upper central incisor contact), which showed statistically significant differences (p < 0.05). Z (R) and Z (L) showed highly significant p values (p < 0.01), while intraobserver variations of points on 3D CT ranged from 0 to 0.6 mm, with the variations less than 0.5 mm for most of the points. The only statistically significant difference between the two investigators was observed for measuring of the left lower molar (LML) (p < 0.05).

Interobserver variations of points between first and second tracings defined on PA Ceph were less than 1.5 mm for all the points. No statistically significant differences were observed with any of the points. Interobserver variations of points between first and second tracings defined on 3D CT were small compared with frontal cephalograms and no statistically significant differences were observed with any of the points.

A direct comparison of frontal cephalometry and 3D CT using intraobserver variation for the first observer showed that 3D CT was more reliable than frontal cepha-lometry for 11 points: Or (R), Or (L), ANS, UM (L), LM (L), Me (p < 0.001, very highly significant), MO (L), NC (L), Ag (L) (p < 0.01, highly significant), UM (R) and Ag (R) (p < 0.05, significant). Direct comparison of frontal cephalometry and 3D CT for the second observer showed a similar tendency with 14 points significantly more reliable for 3D CT -Z (R), Or (R), ANS, UM (R), UM (L), A1 (p < 0.001), Z(L), MO (R), MO (L), Or (L), B1, Ag (R) (p < 0.01), J(R) and LM (R) (p < 0.05).

Comparison of frontal cephalometry and 3D CT using interobserver variation for the first tracings showed that 3D CT was more reliable than frontal cephalometry for 12 points Or (R), NC (R) (p < 0.001), MO (R), MO(L), ANS, UM (L), LM (R), Me (p < 0.01), Or (L), NC (L), UM (R) and Ag (R) (p < 0.05). Comparison of frontal cephalometry and 3D CT using interobserver variation for the second tracings showed a similar trend with 3D CT more reliable than frontal cephalometry for 10 points ANS (p < 0.001), MO (R), Or (R), LM (R), Me (p < 0.01), MO(L), NC (R), NC (L), UM (L) and LM (L) (p < 0.05).

While CT scans offer many advantages over conventional radiography, the high radiation dose to patients and the cost of the procedure should be considered. The somatic risks from X-radiation include leukemia, thyroid cancer, bone cancer, esophageal cancer, brain and nervous system cancer, salivary gland cancer, mental retardation and cataract of the eye.

Darendeliler MA et al^25^ compared the radiation doses of facial CT scans with the radiation doses taking a lateral cephalometric radiograph, a panoramic radiograph (OPG), an occlusal film and an intraoral periapical radiograph. The following radiation doses were measured: Maxillo-mandib-ular CT scan 2.1 mSv; maxillary CT scan 1.40 mSv; mandibular CT scan 1.32 mSv; lateral cephalometric radiograph 0.005 mSv; OPG 0.01 mSv; maxillary occlusal 0.007 mSv; intraoral periapical radiograph 0.005 mSv. They concluded that CT scans produce significantly more ionizing radiation than conventional radiographs. This factor should be taken into account when considering a CT scan as an alternative to a survey with conventional radiographs.

Diederichs CG et al^26^ evaluated the feasibility of performing preoperative spiral CT of the maxilla and mandible with a radiation dose similar to that used for conventional panoramic radiography. The skin entrance doses of radiation used for spiral CT (collimation, 1 mm; pitch, 2; tube voltage, 80 kV; tube current, 40 mA) and for panoramic radiography (75 kV, 8 mA, 15 seconds) were measured in one patient by using thermoluminescent dosimeter chips. Results were 0.56, 6, 0.06 mGy for CT and 0.59, 6, 0.04 mGy for radiography. Image quality was adequate for preoperative implant planning. Spiral CT of the mandible and maxilla may, therefore, be feasible with a radiation dose of similar magnitude as that used for conventional panoramic radiography.

## SUMMARY AND CONCLUSION

The present study was conducted to compare the reliability of anatomic cephalometric points obtained from the two modalities―conventional posteroanterior cephalograms and 3D CT of patients with facial asymmetry, by comparing intra- and interobserver variation of points recorded from frontal X-ray to those recorded from 3D CT.

The study has shown that 3D CT is consistently more accurate and reliable than conventional frontal cephalometry. Intraobserver variations of points defined on 3D CT were small compared with frontal cephalograms. The variation was less than 1 mm for most of the points except right and left zygomatico frontal suture, right orbitale, right J point and A1 (upper central incisor contact), which showed statistically significant differences. Intraobserver variations of points on 3D CT were less than 0.5 mm for most of the points. The only statistically significant difference between the two investigators was observed for measuring of the left lower molar.

Interobserver variations of points between first and second tracings defined on PA Ceph were less than 1.5 mm for all the points. No statistically significant differences were observed with any of the points. Interobserver variations of points between first and second tracings defined on 3D CT were small compared with frontal cephalograms and no statistically significant differences were observed with any of the points.

A direct comparison of frontal cephalometry and 3D CT using intraobserver variation for the first observer showed that 3D CT was more reliable than frontal cephalometry for 11 points―Or (R), Or (L), ANS, UM (L), LM (L), Me, MO (L), NC (L), Ag (L), UM (R) and Ag (R). Direct comparison of frontal cephalometry and 3D CT for the second observer showed a similar tendency, with 14 points significantly more reliable for 3D CT―Z (R), Or (R), ANS, UM (R), UM (L), A1, Z(L), MO (R), MO (L), Or (L), B1, Ag (R), J(R) and LM (R).

Comparison of frontal cephalometry and 3D CT using interobserver variation for the first tracings showed that 3D CT was more reliable than frontal cephalometry for 12 points―Or (R), NC (R), MO (R), MO(L), ANS, UM (L), LM (R), Me, Or (L), NC (L), UM (R) and Ag (R). Comparison of frontal cephalometry and 3D CT using inter-observer variation for the second tracings showed a similar trend with 3D CT more reliable than frontal cephalometry for 10 points―ANS, MO (R), Or (R), LM (R), Me, MO (L), NC (R), NC (L), UM (L) and LM (L).

According to J Treil et al, conventional radiography and cephalometry are outdated in orthodontic practice today. According to them, the switch to computerized tomography scanning from conventional X-rays is ineluctable. Unlike conventional X-rays (periapical, panoramic and headfilms), which yield only 1 view for every exposure, a single computerized tomographic radiation exposure can result in many two-dimensional multiplanar reconstructions and three-dimensional views. Selected anatomical structures can be presented in all three dimensions, the advantages of which are almost self-evident. Unfortunately, the cost of a computerized tomography examination is considerable; this means that, at this stage, its use by surgeons and orthodontists is a last resort.
